# VDR Activation Attenuates Renal Tubular Epithelial Cell Ferroptosis by Regulating Nrf2/HO‐1 Signaling Pathway in Diabetic Nephropathy

**DOI:** 10.1002/advs.202305563

**Published:** 2023-12-25

**Authors:** Hui Wang, Xiaoyue Yu, Dongwei Liu, Yingjin Qiao, Jinling Huo, Shaokang Pan, Lijuan Zhou, Rui Wang, Qi Feng, Zhangsuo Liu

**Affiliations:** ^1^ Research Institute of Nephrology, Zhengzhou University The First Affiliated Hospital of Zhengzhou University Zhengzhou 450052 P. R. China; ^2^ Traditional Chinese Medicine Integrated Department of Nephrology The First Affiliated Hospital of Zhengzhou University Zhengzhou 450052 P. R. China; ^3^ Henan Province Research Center for Kidney Disease Zhengzhou 450052 P. R. China; ^4^ Key Laboratory of Precision Diagnosis and Treatment for Chronic Kidney Disease in Henan Province Zhengzhou 450052 P. R. China; ^5^ Blood Purification Center The First Affiliated Hospital of Zhengzhou University Zhengzhou 450052 P. R. China; ^6^ Electron Microscopy Laboratory of Renal Pathology The First Affiliated Hospital of Zhengzhou University Zhengzhou 450052 P. R. China

**Keywords:** diabetic nephropathy (DN), ferroptosis, Nrf2/HO‐1 signaling pathway, renal tubular epithelial cell, vitamin D receptor (VDR)

## Abstract

Diabetic nephropathy (DN) is a serious microvascular complication of diabetes. Ferroptosis, a new form of cell death, plays a crucial role in the pathogenesis of DN. Renal tubular injury triggered by ferroptosis might be essential in this process. Numerous studies demonstrate that the vitamin D receptor (VDR) exerts beneficial effects by suppressing ferroptosis. However, the underlying mechanism has not been fully elucidated. Thus, they verified the nephroprotective effect of VDR activation and explored the mechanism by which VDR activation suppressed ferroptosis in *db/db* mice and high glucose‐cultured proximal tubular epithelial cells (PTECs). Paricalcitol (PAR) is a VDR agonist that can mitigate kidney injury and prevent renal dysfunction. PAR treatment could inhibit ferroptosis of PTECs through decreasing iron content, increasing glutathione (GSH) levels, reducing malondialdehyde (MDA) generation, decreasing the expression of positive ferroptosis mediator transferrin receptor 1 (TFR‐1), and enhancing the expression of negative ferroptosis mediators including ferritin heavy chain (FTH‐1), glutathione peroxidase 4 (GPX4), and cystine/glutamate antiporter solute carrier family 7 member 11 (SLC7A11). Mechanistically, VDR activation upregulated the NFE2‐related factor 2/heme oxygenase‐1 (Nrf2/HO‐1) signaling pathway to suppress ferroptosis in PTECs. These findings suggested that VDR activation inhibited ferroptosis of PTECs in DN via modulating the Nrf2/HO‐1 signaling pathway.

## Graphical abstract

VDR activation by paricalcitol (PAR) alleviates diabetic kidney injury by inhibiting renal tubular epithelial cell ferroptosis through regulating Nrf2/HO‐1 signaling pathway. During this process, the activated VDR/Nrf2/HO‐1 signaling pathway can significantly decrease the iron content and MDA generation, increase GSH level, reduce the expression of TFR‐1, and enhance the expression of FTH‐1, GPX4, and SLC7A11.

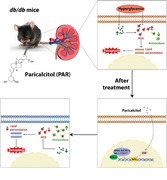

## 1 Introduction

Diabetic nephropathy (DN) is one of the most common microvascular complications of diabetes mellitus (DM) and has become the major cause of kidney failure worldwide.^[^ ^1^, ^2^ ^]^ However, there are few established options for DN patients, including angiotensin converting enzyme inhibitors (ACEIs), angiotensin II receptor antagonists (ARBs), and sodium‐dependent glucose transporter 2 (SGLT‐2) inhibitors,^[^ ^2^, ^3^ ^]^ with unsatisfactory therapeutic efficacy. The pathogenesis of DN involves many aspects, and the “glomerulocentric view” has been dominant for a long time. However, increasing evidence has shown that renal tubular injury may occur earlier than glomerular injury. Changes in mitochondrial dynamics in proximal tubular epithelial cells (PTECs) were observed before the occurrence of proteinuria excretion and renal histological defects in diabetic rats.^[^ ^4^ ^]^ Hasegawa et al. discovered that nicotinamide mononucleotide from PTECs diffused back to the glomerulus to affect podocyte function.^[^ ^5^ ^]^ In addition, renal tubular atrophy and interstitial fibrosis lead to renal dysfunction by secreting inflammatory cytokines and profibrotic molecules, whatever in DN patients with proteinuria or non‐proteinuria.^[^ ^6^, ^7^ ^]^ Therefore, the severity of renal tubular lesions seems strongly connected with the progression of DN.

Ferroptosis, a distinct nonapoptotic type of regulated cell death, depends on intracellular iron overload and is distinguished by the increase of lipid peroxides.^[^ ^8^ ^]^ Recently, many studies have demonstrated that the initiation and development of DN are influenced by the ferroptosis of PTECs.^[^ ^9^, ^10^, ^11^ ^]^ Ferrostatin‐1 (Fer‐1), a synthetic antioxidant compound, can inhibit ferroptosis by reducing reactive oxygen species (ROS) levels, removing cellular unstable iron, and depleting lipid peroxides.^[^ ^12^ ^]^ Li et al. demonstrated that Fer‐1 could alleviate renal tubular damage in type 2 diabetic mice.^[^ ^9^ ^]^ Nevertheless, the application of Fer‐1 is restricted due to its low natural stability in vivo.^[^ ^13^ ^]^ Therefore, it is essential to develop a novel and feasible ferroptosis inhibitor for DN treatment.

Recently, Hu et al. reported that the vitamin D receptor (VDR) might be a transcription factor related to ferroptosis in DN.^[^ ^14^ ^]^ Accumulating evidence has revealed the nephroprotective effect of VDR activation, including immune regulation, inflammatory inhibition and fibrosis alleviation.^[^ ^15^, ^16^, ^17^ ^]^ Paricalcitol (PAR) is an active form of VD for the treatment of kidney diseases.^[^ ^18^ ^]^ A clinical trial found that DN patients treated with PAR generated a reduction in albuminuria excretion.^[^ ^19^ ^]^ Despite the beneficial effects being extensively documented, it is still unclear how VDR activation affects ferroptosis in DN. As reported, VDR activation could inhibit ferroptosis of PTECs in acute kidney injury (AKI) induced by cisplatin.^[^ ^20^ ^]^ Therefore, it is worth exploring the role of VDR activation in the ferroptosis of PTECs under diabetic conditions.

In the present study, we aimed to explore the nephroprotective effects of VDR activation on high glucose‐cultured PTECs and DN mouse models and reveal its underlying mechanisms. Our findings strongly confirmed that VDR activation attenuated diabetic kidney injury by inhibiting ferroptosis, in which the NFE2‐related factor 2 (Nrf2)/heme oxygenase‐1 (HO‐1) signaling pathway played a significant role. Overall, this study proposed a new perspective for investigating the potential of VDR activation in DN treatment.

## 2 Experimental Section

### 2.1 Animal Model and Experimental Groups

All operations were carried out in accordance with the National Institutes of Health (NIH) guidelines for the Care and Use of Laboratory Animals^[^ ^21^ ^]^ and under the approval offered by the Ethics Committee of The First Affiliated Hospital of Zhengzhou University (Ethics Approval No. ZZU‐LAC20201013[09]).

Eight‐week‐old male *db/db* mice and control *db/m* littermates, on a C57BL/KsJ background, were acquired from GemPharmatech (Nanjing, Jiangsu, China) and fed under SPF conditions in the Laboratory Animal Center of Zhengzhou University (Zhengzhou, Henan, China). After 2 weeks of adaptive rearing, *db/m* mice were considered as a) control group (*db/m*, n = 6). Four groups of the *db/db* mice were created at random: b) DN group (*db/db*, *n* = 6); c) DN+Vehicle group (*db/db*+Vehicle, 1% DMSO, *n* = 6); d) DN+Fer‐1 group (*db/db*+Fer‐1, 5 mg kg^−1^ dissolved in 1% DMSO, *n* = 6); and (e) DN+PAR group (*db/db*+PAR, 0.1 µg kg^−1^ dissolved in 1% DMSO, *n* = 6). DMSO, Fer‐1, and PAR received intraperitoneal injection once daily for five consecutive days 1 week. Fer‐1 (HY‐100579) and PAR (HY‐50919) were obtained from MedChemExpress (Shanghai, China). Mouse body weight and blood glucose were measured biweekly and collected urine every 2 weeks for subsequent analysis of urine albumin‐to‐creatinine ratio (uACR). After 10 weeks of treatment, all mice were euthanized and kidney tissues were removed and stored for further research.

### 2.2 Renal Histology and Immunohistochemistry Staining

The preserved kidney tissues were fixed in formalin, paraffin‐embedded, and cut into 3 µm‐thick slices. The samples were deparaffinized and hydrated, then further dyed for PAS via periodic acid oxidation and Schiff's reagent staining. After washing in tap water, the cell nuclei were stained with hematoxylin. Alternatively, hematoxylin and eosin were used to stain the slices for HE staining. Finally, the slices were mounted with neutral gum. For immunohistochemistry analysis, the kidney tissue sections were dewaxed and dehydrated and then incubated in 3% H_2_O_2_. After washing and blocking, the slices were incubated with the primary and secondary antibodies in turn. All sections were colored by DAB staining and imaged through a light microscope (Nikon, Tokyo, Japan). The primary antibodies were as follows: kidney injury molecule ‐1 (Kim‐1, 1:100, AF1817, R&D Systems, Minnesota, MN, USA), glutathione peroxidase 4 (GPX4, 1:100, ab125066, Abcam, Cambridge, UK), ferritin heavy chain 1 (FTH‐1, 1:100, ab65080, Abcam), cystine/glutamate antiporter solute carrier family 7 member 11 (SLC7A11, 1:200, 26864‐1‐AP, Proteintech, Wuhan, Hubei, China), Nrf2 (1:200, 16396‐1‐AP, Proteintech), HO‐1 (1:200, 10701‐1‐AP, Proteintech), transferrin receptor 1 (TFR‐1, 1:100, sc65882, Santa Cruz Biotechnology, CA, USA) and VDR (1:100, sc13133, Santa Cruz Biotechnology).

### 2.3 Prussian Blue Staining

According to the manufacturer's instructions of Prussian blue staining kit (G1424, Solarbio, Beijing, China), the above specimens were deparaffinized, hydrated, and incubated with a 1:1 mixture of hydrochloric acid at a low concentration and 2% potassium ferrocyanide. Then, the slides were rinsed with double‐distilled water three times, and the nuclei were stained with a 0.1% nuclear solid red solution. The sealed sections were viewed to capture the pictures under a light microscope (Nikon).

### 2.4 Cell Culture and RNA Interference

An immortalized human proximal renal tubular epithelial cell line (HK‐2) was purchased from QuiCell (Shanghai, China) and cultured in DMEM plus F12 medium (1:1, Gibco, Gaithersburg, MD, USA) containing 10% fetal bovine serum (Gibco) and 1% penicillin/streptomycin (Gibco) at 37 °C in a humidified atmosphere of 5% CO2. Cells were incubated for 48 h under normal glucose (NG, 5.6 mm glucose), high mannitol (HM, 5.6 mm glucose plus 24.4 mm mannitol), and high glucose (HG, 30 mM glucose) conditions when they reached ≈40–50% confluence. Meanwhile, the HG group was divided into a blank control group (HG), a positive control group (HG+Fer‐1, HG plus 1 µM Fer‐1), and a treatment group (HG+PAR, HG plus 0.1 µM PAR). Trigonelline (Trig, 100 µM), an efficient Nrf2 inhibitor purchased from MedChemExpress, was added to the cells 30 min beforehand.

HK‐2 cells were transfected with Nrf2 small interfering RNA (siRNA; forward strand, 5′‐GACAGAAGUUGACAAUUAUdTdT‐3′; reverse strand, 5′‐AUAAUUGUCAACUUCUGUCdTdT‐3′) or a negative control (Hanbio Biotechnology, Shanghai, China) by using Lipofectamine 3000 (Invitrogen, Carlsbad, CA, USA) according to the manufacturer's guidelines. Cells were collected for subsequent assays at 48 h after transfection.

### 2.5 Cell Viability Detection

Cell viability was measured by using the Cell Counting Kit‐8 (CK04, Dojindo, Kumamoto, Japan) following the manufacturer's instructions. A total of 1 × 10^3^ cells were seeded into each well of a 96‐well plate. Then, PAR or Fer‐1 were added to the cells at different concentrations. At 48 h, the cells were incubated with the working reagent for 2 h at 37 °C. Finally, the absorbance of each well at 450 nm was detected on a microplate reader (Molecular Devices, Shanghai, China).

### 2.6 Detection of ROS Generation

ROS Detection Reagents (MP36103, Invitrogen) were used to measure intracellular ROS generation. After 48 h of stimulation, cells growing in a 24‐well plate with cover slips were incubated with 20 µm carboxy‐H2DCFDA working solution and 20 µm Hoechst at 37 °C for 10 min from light. In the next few hours, the results were presented using a confocal microscope (CarlZeiss, Oberkochen, Germany).

### 2.7 Immunofluorescence Staining

HK‐2 cells were fixed with 4% paraformaldehyde for 30 min, permeabilized with 0.5% Triton X‐100 for 10–30 min at room temperature, blocked with 3% bovine serum albumin, and then incubated with primary antibodies overnight at 4 °C. Antibodies against SLC7A11 (1:250, ab37185), TFR‐1 (1:250, ab214039) and Nrf2 (1:400, ab137550) were purchased from Abcam; antibodies against GPX4 (1:400, 67763‐1‐AP) and HO‐1 (1:400, 10701‐1‐AP) were obtained from Proteintech; and antibodies against VDR (1:250, sc13133) were acquired from Santa Cruz Biotechnology. Subsequently, covered with Alexa Fluor 488 or 594 goat anti‐mouse or goat anti‐rabbit IgG (1:250, Invitrogen) for 1 h and counterstained with 4′,6‐diamidino‐2‐phenylindole (DAPI, Vector Laboratories, Burlingame, CA, USA), the cells were visualized and analyzed by confocal microscope.

### 2.8 Determination of Iron Content

The ferrous iron levels in the kidney cortex tissues and HK‐2 cells were detected by the Iron Assay Kit (MAK025, Sigma–Aldrich, St. Louis, MO, USA). Ten milligrams of kidney cortex tissues were rapidly homogenized by a tissue homogenizer (Scientz, Ningbo, Jiangsu, China), and cells (5 × 10^6^) grown in a T75 culture flask were collected and homogenized by an ultrasonic cell disrupter (Scientz). Then, the supernatants were collected for the next steps according to the procedure description. Finally, the results were determined by a microplate reader at a wavelength of 593 nm.

### 2.9 Determination of Malondialdehyde (MDA) and Glutathione (GSH) Levels

The MDA and GSH contents in the cortex tissues and HK‐2 cells were examined using the Lipid Peroxidation MDA Assay Kit (S0131, Beyotime, Shanghai, China) and Micro Reduced GSH Assay Kit (BC1175, Solarbio), respectively. Both tests were performed as directed by the manufacturer, and the content of each sample was identified with a microplate reader at the corresponding wavelength (532 nm for MDA and 412 nm for GSH). The MDA and GSH levels were standardized by protein amount in kidney homogenates or cell lysates.

### 2.10 Transmission Electron Microscopy (TEM)

Mice kidney tissue or HK‐2 cells were fixed in glutaraldehyde and 1% osmium acid. Then, the fixed specimens were dehydrated in acetone, soaked and embedded with a mixture of embedding agent and dehydrating agent. The specimens were coated with 812 epoxy resin and polymerized at 60 °C. The ultrathin sections were cut at 60 nm with an ultramicrotome. After being stained with uranyl acetate and lead citrate together, the slices were photographed and analyzed using an electron microscope (Hitachi, Tokyo, Japan).

### 2.11 Quantitative Real‐Time Polymerase Chain Reaction (qRT‐PCR)

Total RNA was extracted from HK‐2 cells using the TRIzol reagent (Invitrogen) following the guidance provided by the manufacturer. After being diluted, RNA was used for reverse transcription with the RevertAid First Strand cDNA Synthesis Kit (Thermo Fisher Scientific, Waltham, MA, USA) to produce cDNA. Each sample was amplified to reflect the expression of the target gene using Maxima SYBR Green qPCR Master Mix (Thermo Fisher Scientific), and the signals were detected by Applied Biosystems QuantStudio 5 (Thermo Fisher Scientific). The primers were designed and synthesized by Sangon Biotechnology (Shanghai, China), and the sequences are listed in **Table** **1**. *ACTB* served as the internal control.

**Table 1:** Primer sequences for qRT‐PCR.

Gene	Forward Primer,5′‐3′	Reverse Primer,5′‐3′
*SLC7A11*	TTACCAGCTTTTGTACGAGTCT	GTGAGCTTGCAAAAGGTTAAGA
*GPX4*	ATGGTTAACCTGGACAAGTACC	GACGAGCTGAGTGTAGTTTACT
*TFRC*	TGAACCAATACAGAGCAGACAT	GTTTTCTCAGCATTCCCGAAAT
*FTH1*	GCCATCAACCGCCAGATCAACC	ATTCAGCCCGCTCTCCCAGTC
*AIFM2*	GCAGACGGACAAAGGCACAGAG	GGCACAGTCACCAATGGCGTAG
*VDR*	AAAGGTCATTGGCTTTGCTAAG	CTTGACTTCAGCAGTACGATCT
*NFE2L2*	AGTCCAGAAGCCAAACTGACAGAAG	GGAGAGGATGCTGCTGAAGGAATC
*HMOX1*	TGCCAGTGCCACCAAGTTCAAG	TGTTGAGCAGGAACGCAGTCTTG
*ACTB*	GCACTCTTCCAGCCTTCCTTCC	GCGGATGTCCACGTCACACTTC

### 2.12 Western Blotting Analysis

Murine renal tissue and HK‐2 cells were lysed by radioimmunoprecipitation assay (RIPA) lysis buffer (Solarbio), and total proteins were extracted from cells or tissue supplemented with protease inhibitors (CWBio, Beijing, China) and phosphatase inhibitors (CWBio). After quantification by a BCA protein assay kit (Solarbio), proteins were set apart by sodium dodecylsulfate polyacrylamide gel electrophoresis and transferred to nitrocellulose membranes (Millipore, Billerica, MA, USA). The membranes were blocked in 5% nonfat milk in TBST buffer (Tris buffer saline containing 0.1% Tween‐20) for 2 h at room temperature, incubated with primary antibodies overnight at 4 °C and secondary antibodies for 1 h at room temperature, and developed using an ECL system (Tanon, Shanghai, China). Antibodies against VDR (1:1000, ab109234), GPX4 (1:1000, ab125066), SLC7A11 (1:1000, ab175186), TFR‐1 (1:1000, ab84036), and Kim‐1 (1:1000, ab78494) were purchased from Abcam; antibodies against ferroptosis suppressor protein 1 (FSP1, 1:1000, 20886‐1‐AP), and Neutrophil gelatinase‐associated lipocalin (NGAL, 1:1000, 26991‐1‐AP), Nrf2 (1:1000, 16396‐1‐AP) and HO‐1 (1:1000, 10701‐1‐AP) were acquired from Proteintech; antibodies against FTH‐1 (1:1000, DF6278) were purchased from Affinity Biosciences (Liyang, Jiangsu, China); and antibodies against β‐actin (1:5000, AP0060) were obtained from Bioworld (Nanjing, Jiangsu, China). Secondary antibodies were acquired from Dingguo (1:5000, Beijing, China).

### 2.13 Statistical Analyses

All statistical analyses were performed using Prism 8.0 software (GraphPad, La Jolla, CA, USA). All data are expressed as the mean ± standard deviation (SD). For two‐group comparisons, Student's *t* test was performed. One‐way ANOVA was used to evaluate the data of two or more groups. *p* < 0.05 was considered to present a statistically significant difference.

## 3 Results

### 3.1 VDR Activation Effectively Diminished HG‐Induced HK‐2 Cell Injury

**Figure 1:**
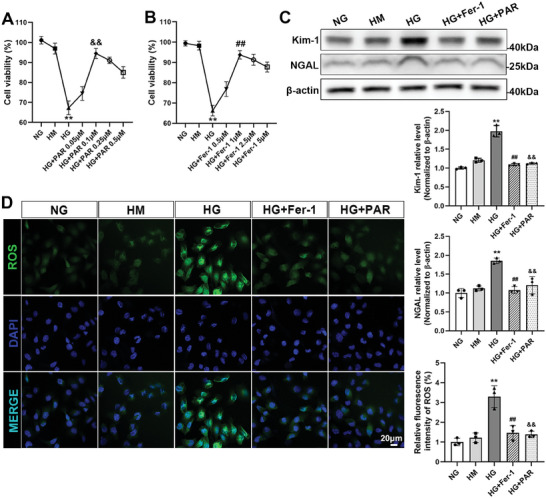
VDR activation alleviated the injury of HK‐2 cells cultured in HG medium. Cell viability was quantified after the cells were cultured in different doses of PAR or Fer‐1 with 30 mm glucose medium for 48 h (A,B). The expression of Kim‐1 and NGAL was examined by western blotting analysis, followed by densitometric analysis of the blots. β‐actin served as a loading control (C). Representative micrographs of immunofluorescence staining for ROS (green) in different groups. Quantitative statistical analysis of the fluorescence intensity of ROS per fixed field was performed. Scale bar = 20 µm (D). Each bar represents the mean ± SD of the data derived from three independent experiments (*n* = 3). ^**^ *p* < 0.01 versus NG group; ^##^ *p* < 0.01 versus HG group; ^&&^ *p* < 0.01 versus HG group.

To detect the impact of VDR activation on renal tubular injury under hyperglycemic conditions, HK‐2 cells were exposed to 30 mm glucose (HG) medium and treated with PAR or Fer‐1 at different concentrations. In addition, cells incubated in medium with 5.6 mm glucose (NG) and 5.6 mm glucose plus 24.4 mM mannitol (HM) were performed as control groups. After 48 h, we used the CCK‐8 assay kit to detect cell viability. The cell viability of the HG group was substantially lower than that of the NG group. PAR treatment markedly improved cell viability under HG conditions, and the optimum was observed at a concentration of 0.1 µM (**Figure** **1**A). Furthermore, to investigate whether the HG‐induced decline in cell viability was related to ferroptosis, Fer‐1 was applied to the cells at different concentrations. The outcomes demonstrated that Fer‐1 treatment also increased cell viability, peaking at a concentration of 1 µm (Figure 1B). Meanwhile, western blotting results revealed that the protein expression levels of Kim‐1 and NGAL in the HG group were significantly higher than that in the control groups, however, this could be reversed by Fer‐1 or PAR treatment (Figure 1C). In the immunofluorescence assay of intracellular ROS production, the fluorescence intensity of the HG group was higher compared to the control groups, but Fer‐1 and PAR treatment effectively reduced ROS generation under HG conditions (Figure 1D).

### 3.2 VDR Activation Inhibited Ferroptosis in HG‐Cultured HK‐2 Cells

**Figure 2:**
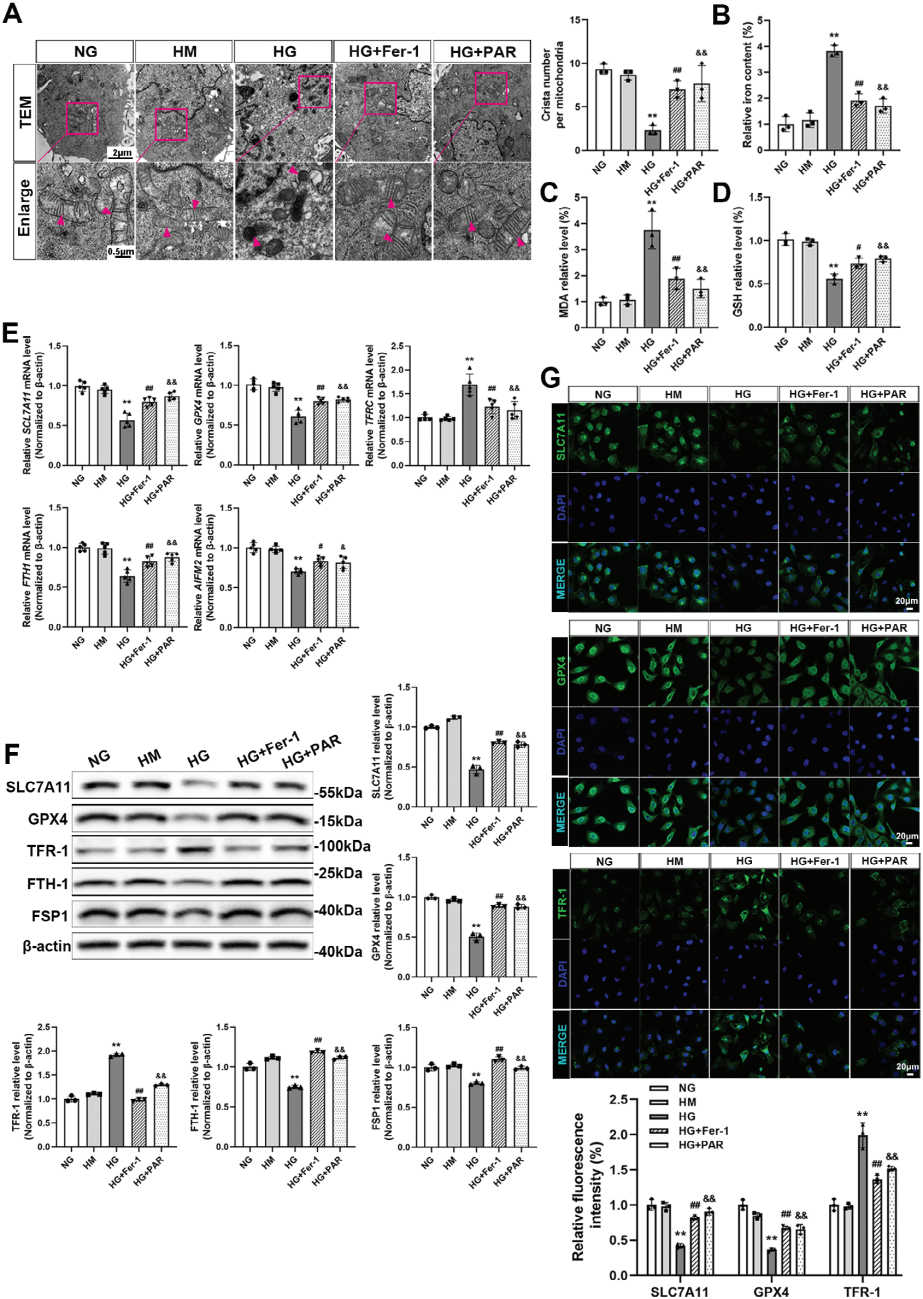
VDR activation suppressed ferroptosis in HG‐cultured HK‐2 cells. The mitochondrial morphology of HK‐2 cells was visualized by TEM. The arrow indicates microscopic changes in mitochondria. Absolute counting of the number of cristae per mitochondria. Scale bar = 2 or 0.5 µm (A). Quantitative analysis of iron (B), MDA (C), and GSH (D) levels in each cell group. The relative mRNA levels of *SLC7A11*, *GPX4*, *TFRC*,*F* *TH1*, and *AIFM2* in different groups of HK‐2 cells were determined by qRT‐PCR. *ACTB* served as a loading control (E). Western blotting was applied to detect the protein expression levels of SLC7A11, GPX4, TFR‐1, FTH‐1, and FSP1, followed by densitometric analysis of the blots. β‐actin served as a loading control (F). Immunofluorescence staining and fluorescence intensity analysis of SLC7A11, GPX4, and TFR‐1 in different groups of HK‐2 cells as indicated. Scale bar = 20 µm (G). Each bar represents the mean ± SD of the data derived from three independent experiments (*n* = 3). ^**^ *p* < 0.01 versus NG group; ^#^ *p* < 0.05, ^##^ *p* < 0.01 versus HG group; ^&^ *p* < 0.05, ^&&^ *p* < 0.01 versus HG group.

TEM directly reflects the degree of ferroptosis through morphological alterations in mitochondria. In the HG group, the mitochondria shrank obviously, and their cristae almost disappeared. However, in the Fer‐1 or PAR treatment groups, the number of mitochondrial cristae increased, and reductions in mitochondrial volume were mitigated (**Figure** **2**A). Additionally, the iron content of HK‐2 cells was tested using an iron assay kit. As shown in Figure 2B, HG treatment induced an over threefold increase in iron content. Conversely, Fer‐1 or PAR treatment noticeably terminated the high level of iron content. Figure 2C shows that the MDA content in the HG group increased more than three times but was mitigated after receiving Fer‐1 or PAR treatment. Figure 2D shows that Fer‐1 or PAR treatment apparently recovered the reduced GSH content induced by HG. Additionally, the qRT‐PCR results showed that the mRNA expression levels of *SLC7A11, GPX4, FTH1*, and *AIFM2* (the gene name for FSP1) were decreased in the HG group but reversed by Fer‐1 or PAR treatment. However, *TFRC* (the gene name for TFR‐1) expression was completely reversed (Figure 2E). Consistently, western blotting analysis of different cell specimens with the respective densitometric measurements confirmed that the expression of ferroptosis‐related proteins could be modulated by Fer‐1 or PAR treatment (Figure 2F). In addition, as corroborated by immunofluorescence analysis, SLC7A11 and GPX4 expression were increased, while TFR‐1 expression was downregulated following treatment with Fer‐1 or PAR (Figure 2G).

### 3.3 VDR Activation Ameliorated Kidney Injury in DN Mice

**Figure 3:**
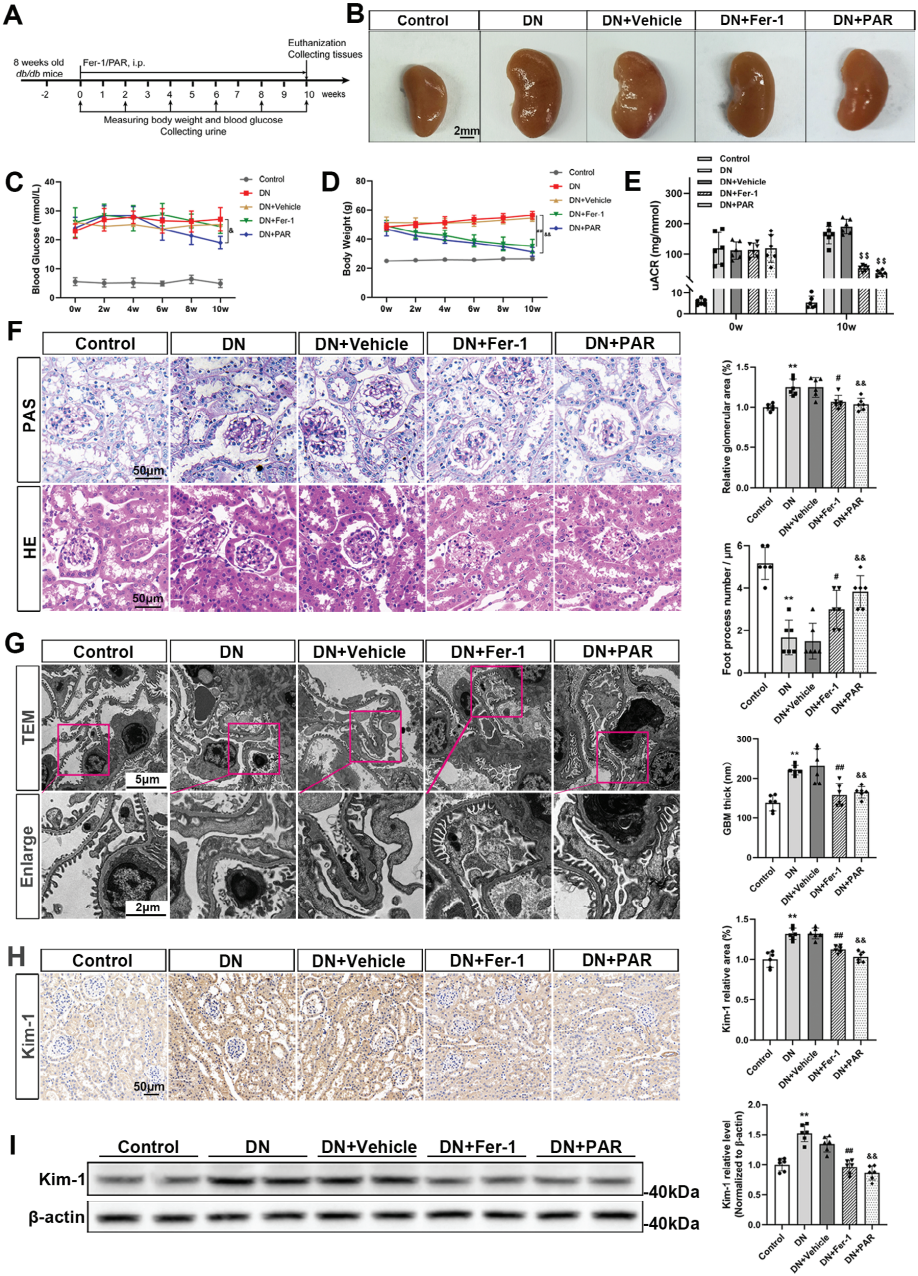
VDR activation attenuated renal dysfunction in DN mice. The animal experiment was designed as shown in the schematic diagram. *db/db* mice aged 10 weeks were randomized to receive a daily i.p. injection of Fer‐1 (5 mg kg^−1^ per day), PAR (0.1 µg kg^−1^ per day) or an equal amount of vehicle for 10 weeks (A). Changes in the renal volume of mice in different groups at the endpoint (B). Blood glucose levels (C) and body weights (D) of mice were examined at the indicated timepoints. Mouse uACRs were tested at 0 and 10 weeks (E). PAS staining and HE staining of murine kidney tissues in different groups. Semiquantitative analysis of the glomerular area in different groups. Scale bar = 50 µm (F). Representative TEM images of glomerular lesions in *db/db* mice and comparative analysis of the GBM foot process number per micrometer and the thickness of the GBM. Scale bar = 5 or 2 µm (G). Immunohistochemical staining and semi‐quantification of Kim‐1 expression in mouse kidney specimens. Scale bar = 50 µm (H). Western blotting analysis of Kim‐1 expression in renal tissue lysates and the densitometric analysis of the blots. β‐actin served as a loading control (I). Each bar represents the mean ± SD of the data derived from six independent experiments (*n* = 6). ^**^ *p* < 0.01 versus Control group; ^#^ *p* < 0.05, ^##^ *p* < 0.01 versus DN group; ^&^ *p* < 0.05, ^&&^ *p* < 0.01 versus DN group; ^$$^ *p* < 0.01 versus baseline of each group.

As depicted in **Figure** **3**A, we established the murine models by employing type 2 diabetes *db/db* mice to investigate the effect of VDR activation on renal function in DN. The *db/m* mice were used as controls. Vehicle, Fer‐1 and PAR were injected for 10 weeks, and blood and urine were collected every 2 weeks to test the blood glucose and uACR values. After 10 weeks of treatment, the renal volume of DN or DN+Vehicle group mice was significantly increased, while Fer‐1 or PAR treatment significantly attenuated kidney hypertrophy in DN mice (Figure 3B). Compared with the control group, the blood glucose levels and body weight of DN mice were significantly increased. As the treatments continued in DN mice, PAR markedly reduced body weight and blood glucose levels; however, Fer‐1 therapy seemed ineffective in lowering blood glucose in the DN group (Figure 3C,D). Fer‐1 or PAR treatment effectively ameliorated albuminuria, as quantified by the uACR value (Figure 3E). As shown by histologic signs of renal damage in DN mice, such as hypertrophied glomeruli, accumulated mesangial matrix, fusion of podocyte foot process, and thickened glomerular basement membrane (GBM), Fer‐1 or PAR treatment could partly relieve diabetic kidney injury (Figure 3F,G). Immunohistochemistry staining and western blotting analysis of renal tissues showed that the expression of Kim‐1 was enhanced in DN mice and reversed by Fer‐1 or PAR treatment (Figure 3H,I).

### 3.4 VDR Activation Suppressed Ferroptosis in DN Mice

**Figure 4:**
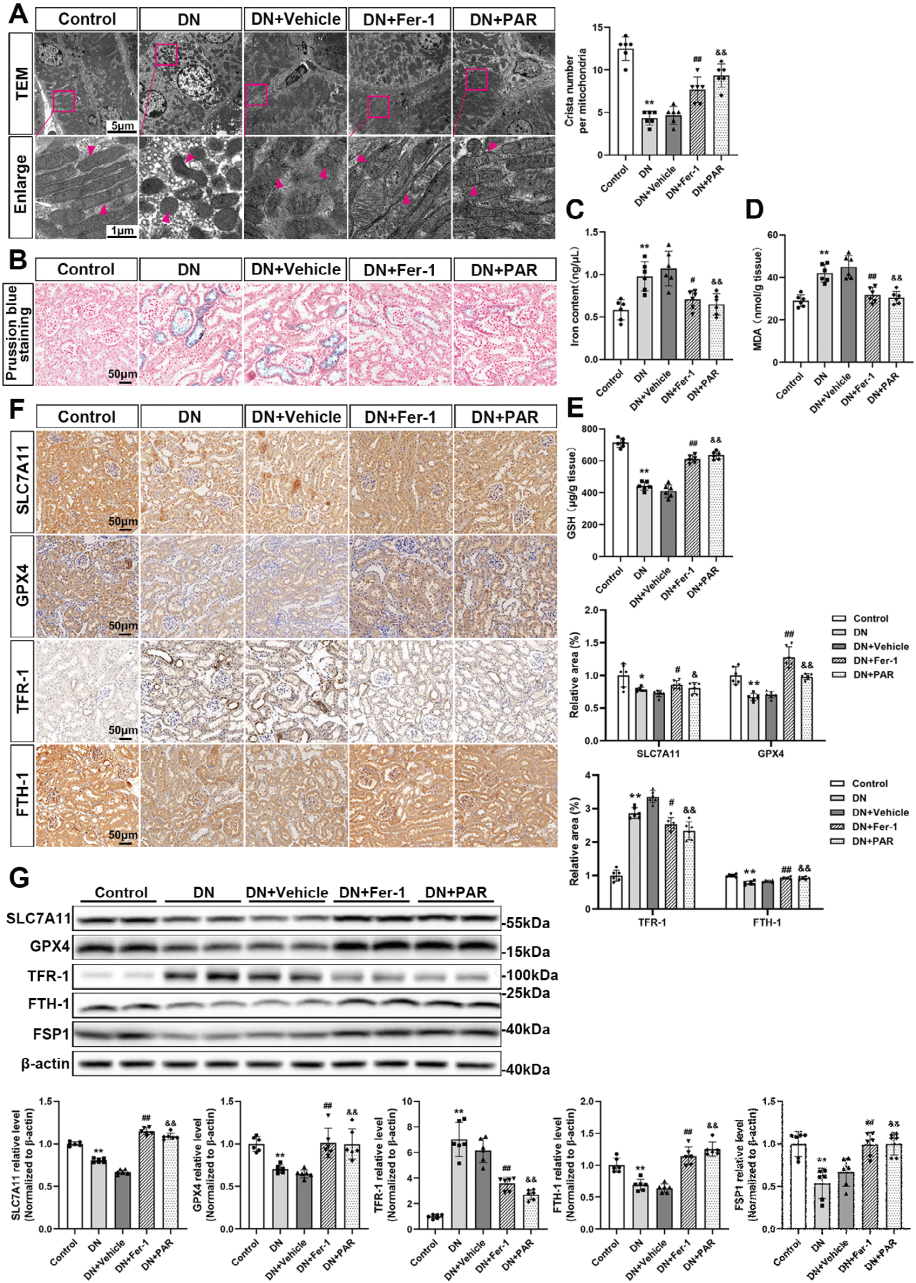
VDR activation inhibits ferroptosis in DN mice. Mitochondrial morphology in renal tissues of different groups as indicated in TEM images. The arrow indicates microscopic changes in mitochondria. Absolute counting of the number of cristae per mitochondria. Scale bar = 5 or 1 µm (A). Prussian blue staining of mouse kidney tissues in each group, scale bar = 50 µm (B). The iron (C), MDA (D), and GSH (E) contents in mouse kidney tissue lysates. Representative microscopic images are shown for immunohistochemistry staining for SLC7A11, GPX4, TFR‐1, and FTH‐1 of kidney tissues in different groups, followed by densitometric analysis of the positive area. Scale bar = 50 µm (F). Western blotting analysis of SLC7A11, GPX4, TFR‐1, FTH‐1, and FSP1 in different groups of renal tissue and densitometric analysis of the blots. β‐actin served as a loading control (G). Each bar represents the mean ± SD of the data derived from six independent experiments (*n* = 6). ^*^ *p* < 0.05, ^**^ *p* < 0.01 versus Control group; ^#^ *p* < 0.05, ^##^ *p* < 0.01 versus DN group; ^&^ *p* < 0.05, ^&&^ *p* < 0.01 versus DN group.

To investigate the beneficial effect of PAR on ferroptosis in vivo, kidney tissues were further examined by TEM. As shown in **Figure** **4**A, the ultrastructural analysis showed that the mitochondria in the DN group had unique morphological changes of ferroptosis, including shrunken mitochondria and reduction or even disappearance of mitochondrial cristae, which demonstrated that ferroptosis occurred in DN mice. After treatment with Fer‐1 or PAR, the mitochondrial morphological changes in DN mice were obviously mitigated. In addition, Prussian blue staining showed that excess iron was deposited around the renal tubules in DN mice, while Fer‐1 or PAR treatment alleviated iron accumulation (Figure 4B). Compared with the DN group, the iron content of renal tissue was decreased by treatment with Fer‐1 or PAR (Figure 4C), and the MDA level was also alleviated by Fer‐1 or PAR (Figure 4D). The GSH levels of the treatment groups were also increased compared with the DN group (Figure 4E). Moreover, the results of renal tissue immunohistochemistry staining showed the protein expression levels of SLC7A11, GPX4, and FTH‐1 were manifestly increased, and the level of TFR‐1 was decreased in the Fer‐1 and PAR groups compared with the DN group (Figure 4F). In line with the in vitro western blotting findings, the expression levels of SLC7A11, GPX4, FTH‐1 and FSP1 were enhanced in Fer‐1‐ or PAR‐treated mice, but TFR‐1 expression was attenuated (Figure 4G).

### 3.5 VDR Activation Promoted the Expression of Nrf2 and HO‐1 in HG‐Cultured HK‐2 Cells

**Figure 5:**
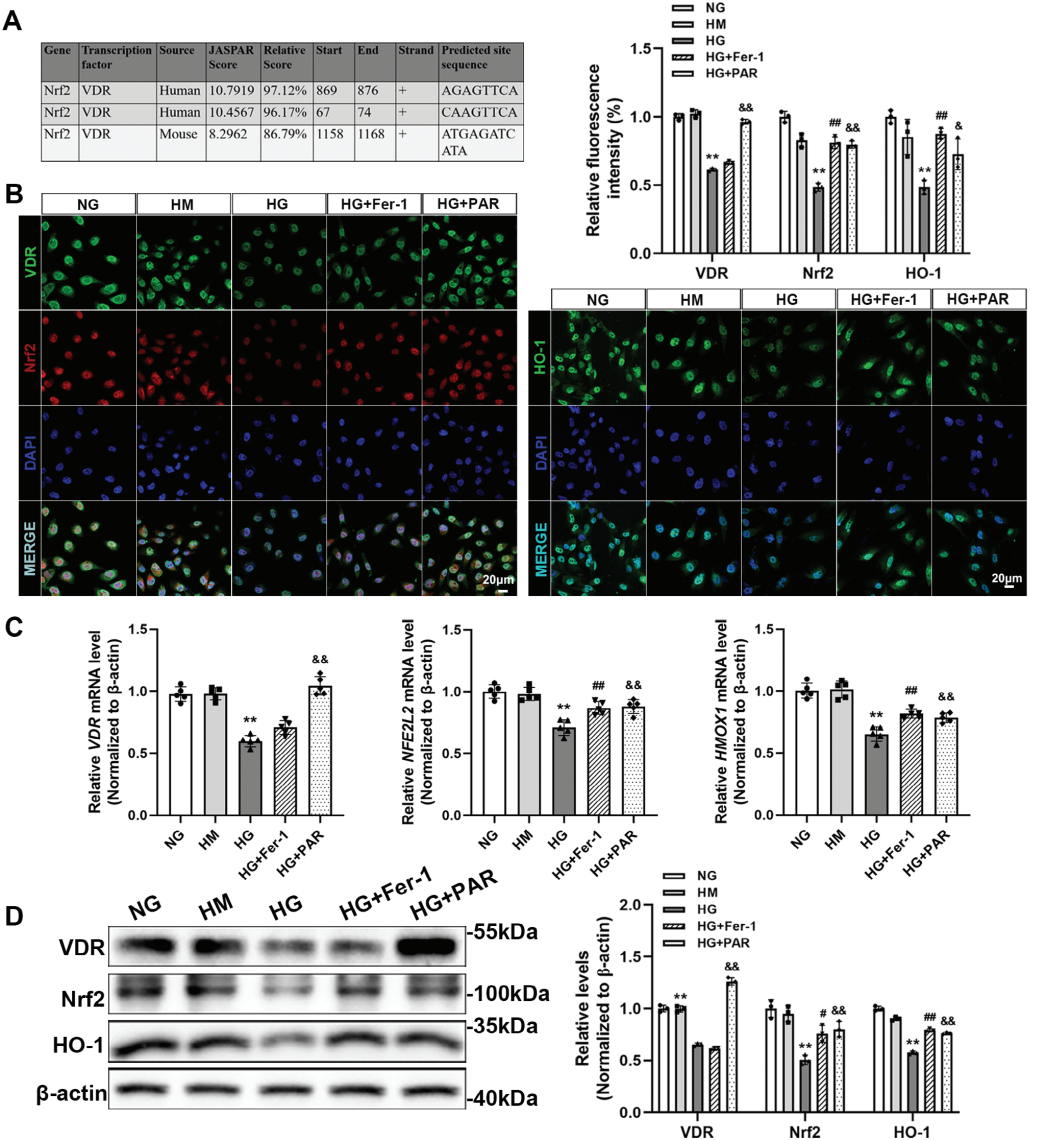
VDR activation upregulated the expression of Nrf2 and HO‐1 in HG‐cultured HK‐2 cells. Predicted VDR binding sites in human and mouse Nrf2 gene promoter regions by JASPAR online analysis (A). Immunofluorescence staining was used to determine the expression and localization of VDR, Nrf2, and HO‐1. Quantitative analysis of the fluorescence intensity per fixed field. Scale bar = 20 µm (B). The relative mRNA levels of *VDR*, *NFE2L2*, and*HMOX1* of HK‐2 cells determined by qRT‐PCR. *ACTB* served as a loading control (C). Western blotting was performed to measure the protein levels of VDR, Nrf2, and HO‐1, followed by densitometric analysis of the blots. β‐actin served as a loading control (D). Each bar represents the mean ± SD of the data derived from three independent experiments (*n* = 3). ^**^ *p* < 0.01 versus Control group; ^#^ *p* < 0.05, ^##^ *p* < 0.01 versus DN group; ^&^ *p* < 0.05, ^&&^ *p* < 0.01 versus DN group.

Many studies have reported that the expression of Nrf2 can be modulated by VDR,^[^ ^22^ ^]^ and a recent study suggested that VDR activation could inhibit ferroptosis by mediating Nrf2 activation in APP/PS1 mice.^[^ ^23^ ^]^ To further test the mechanism by which VDR activation regulates Nrf2 in the progression of ferroptosis in DN, we used the JASPAR database (http://jaspar.genereg.net) to predict the binding sites of *VDR* to *NFE2L2* from both human and mouse homologous sequences (**Figure** **5**A). The results showed that the transcriptional regulation of *NFE2L2* by *VDR* probably exists. The immunofluorescence results of cells proved that VDR and Nrf2 were colocalized in the cell nucleus. In addition, PAR upregulated the expression of VDR, and the fluorescence intensity of Nrf2 and HO‐1 was stronger in the Fer‐1 or PAR group than in the HG group (Figure 5B). Results of qRT‐PCR suggested that *VDR* expression was upregulated by PAR treatment compared with the HG group, and Fer‐1 or PAR treatment dramatically upregulated *NFE2L2* and *HMOX1*expression levels (Figure 5C). The changes in protein level, as shown in western blotting analysis, had the same trend as the qRT‐PCR findings (Figure 5D).

### 3.6 VDR Activation Enhanced Nrf2 and HO‐1 Expression in DN Mice

**Figure 6:**
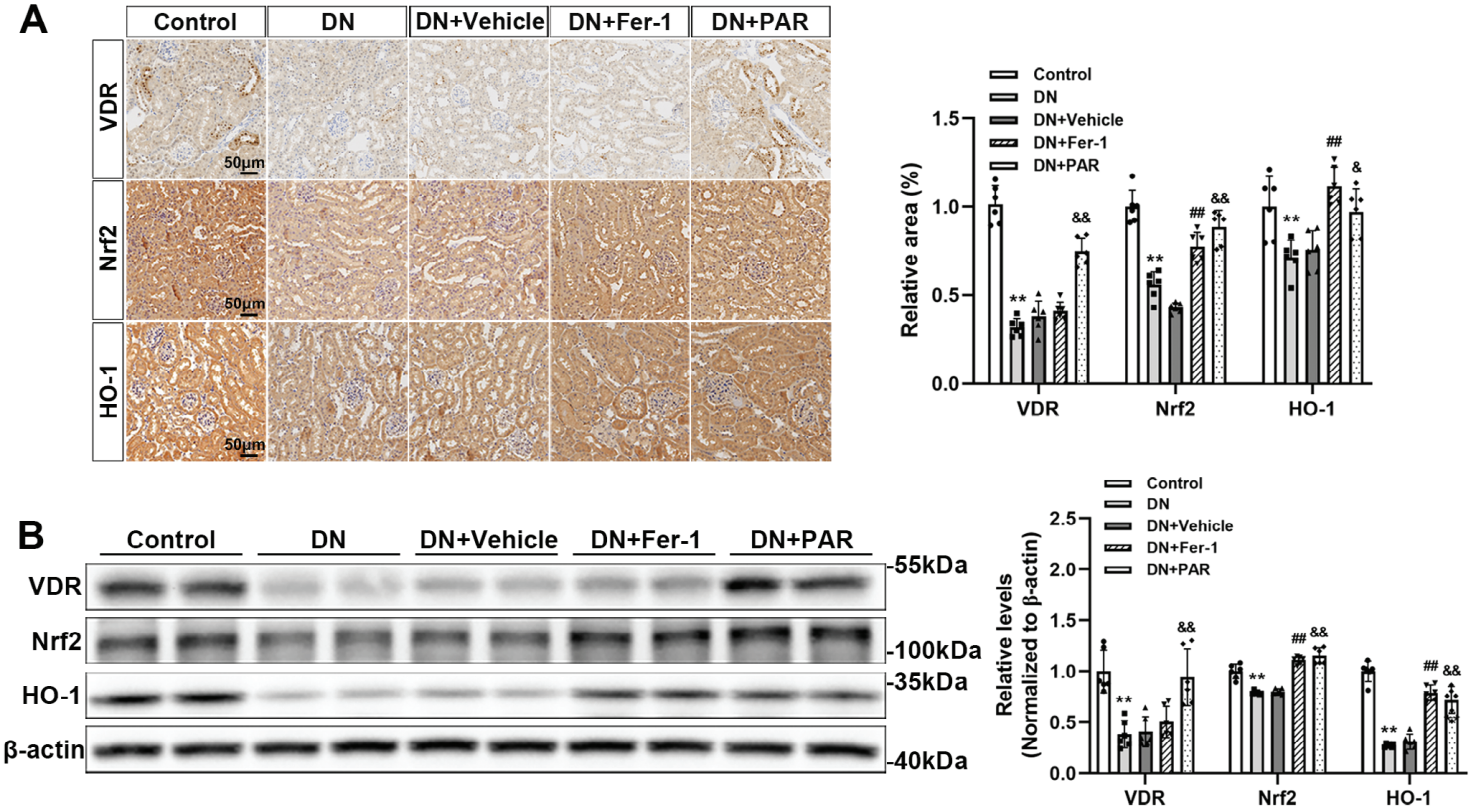
VDR activation upregulated the expression of Nrf2 and HO‐1 in DN mice. Representative microscopic images are shown for immunohistochemistry staining for VDR, Nrf2, and HO‐1 in kidney tissues, followed by densitometric analysis of the positive area. Scale bar = 50 µm (A). Western blotting analysis of VDR, Nrf2, and HO‐1 in renal tissue of different groups and the densitometric analysis of the blots. β‐actin served as a loading control (B). Each bar represents the mean ± SD of the data derived from six independent experiments (*n* = 6). ^**^ *p* < 0.01 versus Control group; ^##^ *p* < 0.01 versus DN group; ^&^ *p* < 0.05, ^&&^ *p* < 0.01 versus DN group.

In keeping with the findings in vitro, immunohistochemistry staining and western blotting results also demonstrated that DN mice had greatly lower protein levels of VDR, Nrf2, and HO‐1. However, with the involvement of PAR, the expression level of VDR approached that in the *db/m* mice, and Fer‐1 or PAR treatment both improved the expression levels of Nrf2 and HO‐1 (**Figure** **6**A,B).

### 3.7 Nrf2 Deficiency Attenuated the Protective Effect of VDR Activation in HG‐Cultured HK‐2 Cells

**Figure 7:**
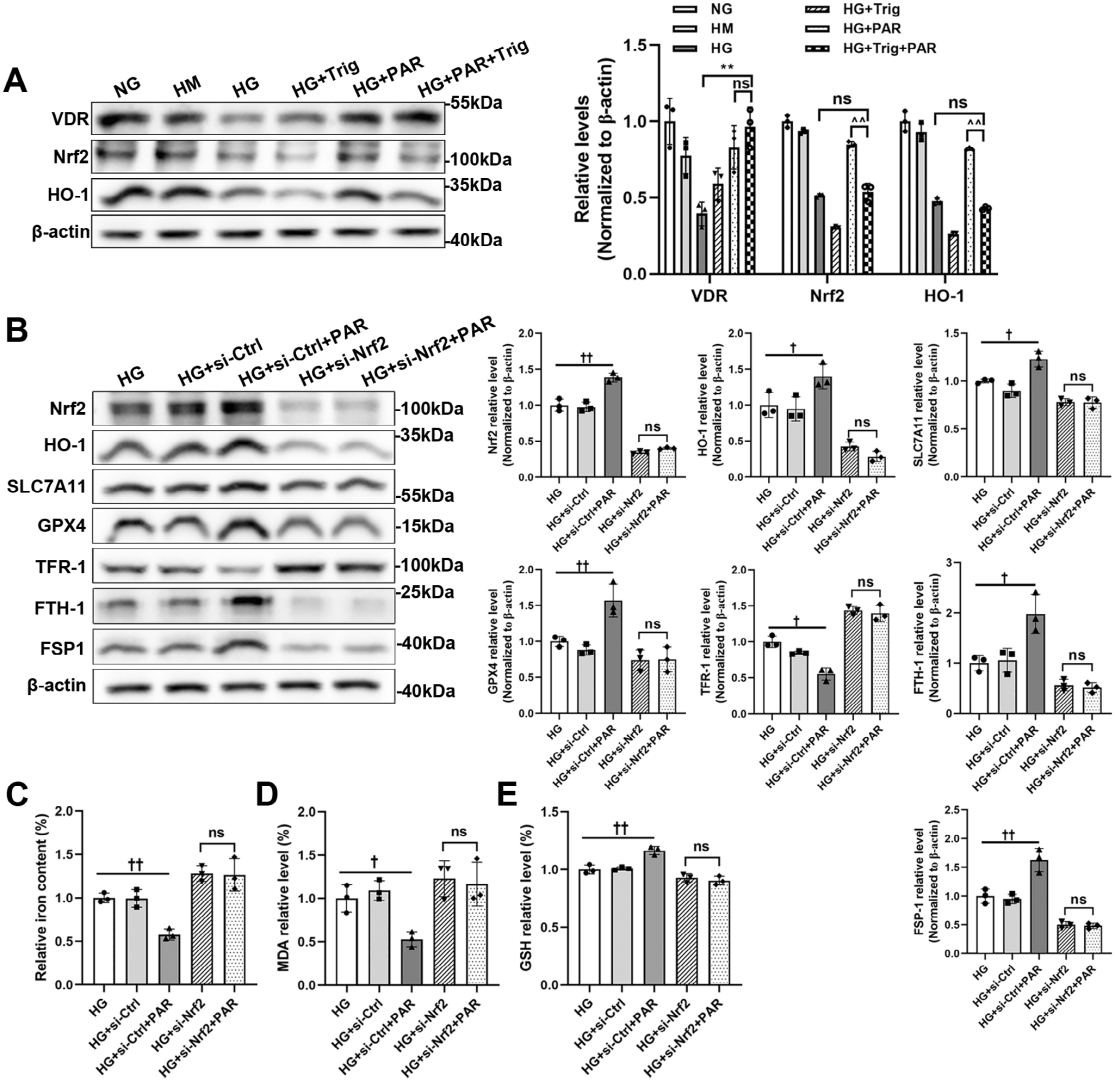
Nrf2 deficiency eliminated the antiferroptotic effect of VDR. Trig was pretreated to HG‐cultured HK‐2 cells for 30 min. Western blotting and related densitometric analysis were applied to detect the expression levels of VDR, Nrf2, and HO‐1. β‐actin served as a loading control(A). HK‐2 cells were transfected with Nrf2‐specific siRNA or negative control siRNA under high glucose conditions. Western blotting was used to detect the expression levels of Nrf2, HO‐1, SLC7A11, GPX4, TFR‐1, FTH‐1, and FSP1 with or without PAR intervention, followed by densitometric analysis of the blots. β‐actin served as a loading control (B). Quantitative analysis of iron (C), MDA (D), and GSH (E) levels in each cell group. Each bar represents the mean ± SD of the data derived from three independent experiments (*n* = 3). ^**^ *p* < 0.01 versus HG group; ^^^^ *p* < 0.01 versus HG+PAR group; ^†^ *p* < 0.05, ^††^ *p* < 0.01 versus HG group and HG+si‐Ctrl group; ns indicates no significance.

Furthermore, to determine whether Nrf2 plays a key role in the process by which VDR regulates ferroptosis, we used trigonelline, a selective inhibitor of Nrf2, to reduce the activated form of Nrf2. According to the findings, the enhanced expression of Nrf2 and HO‐1 induced by PAR in HG‐cultured HK‐2 cells was significantly abolished after treatment with trigonelline (**Figure** **7**A). In addition, HK‐2 cells in HG medium were transfected with *NFE2L2*‐specific siRNA (si‐Nrf2) or negative control siRNA (si‐Ctrl) and treated with or without PAR. As shown in Figure 7B, si‐Nrf2 successfully overrode PAR‐triggered Nrf2 activation. The target protein of Nrf2, HO‐1, was significantly decreased in both si‐Nrf2 groups with or without PAR treatment compared with the siRNA control groups. Furthermore, PAR treatment could regulate the expression of SLC7A11, GPX4, FTH‐1, and TFR‐1 in the si‐Ctrl group but not in the si‐Nrf2 group. Quantitative analysis of iron (Figure 7C) showed that PAR did not change the iron content of the si‐Nrf2 group. Similar to the results of MDA (Figure 7D) and GSH (Figure 7E), there was no significant difference between the HG+si‐Nrf2 group and the HG+si‐Nrf2+PAR group.

## 4 Discussion

DN has been viewed as a clinical challenge for a long time due to its complex pathogenesis and unsatisfactory prognosis. Herein, it is necessary to determine the exact pathophysiology and create effective treatment methods. With the success in the use of SGLT2 inhibitors, the importance of tubular lesions in DN is getting more attention.^[^ ^24^ ^]^ Recent studies have revealed that renal tubule ferroptosis plays an essential role in the development of DN.^[^ ^25^ ^]^ The findings of this study revealed that PAR, an agonist of VDR, could protect against ferroptosis of PTECs and attenuate kidney injury in DN. Furthermore, we utilized a Nrf2‐knockdown cell model to further verify that VDR activation inhibited ferroptosis by regulating the Nrf2/HO‐1 pathway.

Abundant evidence has demonstrated that ferroptosis participates in diabetic kidney injury.^[^ ^9^, ^10^ ^]^ Under hyperglycemic conditions, excess iron promotes the formation of ROS, which are responsible for lipid peroxidation and cell damage, or directly catalyzes the lipid peroxidation by enhancing the activity of lipoxygenases in renal tubular cells.^[^ ^26^, ^27^ ^]^ In addition, suppression of antioxidant systems, including superoxide dismutase, catalase and glutathione peroxidase, contributes to the low efficiency of the elimination of lipid peroxidation.^[^ ^28^ ^]^ Therefore, ferroptotic cell death is triggered and aggravates renal injury. In this study, we observed surplus iron deposits in the kidney tubules, which is consistent with the overexpression of FTH‐1 and downregulation of TFR‐1. The MDA and iron contents were upregulated in HG‐incubated HK‐2 cells and DN murine models. For antioxidation, decreased generation of GSH, improved production of ROS, and lower expression of SLC7A11 and GPX4 were observed under diabetic conditions.

VDR plays an effective role in alleviating kidney injury in DN. Most of the mechanistic studies of VDR activation in DN are related to restoring podocyte autophagy,^[^ ^29^ ^]^ reducing inflammation,^[^ ^15^ ^]^ and suppressing the renin‐angiotensin system (RAS).^[^ ^16^ ^]^ Nevertheless, the role of VDR in PTECs has rarely been explored, and it is elusive. A few studies have proposed that VDR activation exerts a nephroprotective effect in animal models of AKI by regulating the death mode of PTECs.^[^ ^20^ ^]^ A growing body of evidence has confirmed VDR can mediate nongenomic pathways to respond to proximal tubulopathy, such as the AMP‐activated protein kinase (AMPK) and phosphoinositide 3‐kinase (PI3k) pathways.^[^ ^30^, ^31^ ^]^ Our study focused on the early stage of DN, and we demonstrated that VDR activation could improve kidney function by inhibiting ferroptosis. VDR activation mitigated the excretion of urinary protein, reversed the hypertrophy of diabetic kidneys, and alleviated the kidney injury upon diabetic insult in vivo. In addition, it could ameliorate the glycemic level to some degree. The nephroprotective effect of VDR could be multifactorial. It probably depended on the correction of diabetes or the pathway for suppressing ferroptosis. Our present study found that the pathological changes related to ferroptosis were mitigated by the application of PAR in HG‐incubated HK‐2 cells and DN mice, as evidenced by decreased MDA levels and iron content and increased generation of GSH after treatment with PAR. In addition, PAR reversed the expression of proteins and mRNA related to ferroptosis under diabetic conditions, including SLC7A11, GPX4 and FSP1. GPX4 is a core regulator of ferroptosis.^[^ ^32^ ^]^ FSP1 was identified as a ferroptotic regulator by Doll et al., and it could regenerate the reduced form of CoQ10 utilizing nicotinamide adenine dinucleotide phosphate (NADPH). CoQ10 eventually removes lipid peroxides through an oxidation reaction that is independent of GPX4.^[^ ^33^ ^]^ These data suggested that VDR activation increased intracellular antioxidant capacity by upregulating the expression of GPX4 and FSP1.

However, the underlying mechanism of ferroptosis regulated by VDR activation is obscure. An increasing number of studies have identified that ferroptosis is inseparable from oxidative stress^[^ ^34^ ^]^ and that Nrf2 is essential for maintaining cellular redox homeostasis and suppressing ferroptosis.^[^ ^35^ ^]^ The present study supports that PAR shows a potent antioxidant capacity via transcription of Nrf2 signaling. A recent study discovered that 1,25‐dihydroxyvitamin D exerts an antioxidant role by activating Nrf2 signaling,^[^ ^36^ ^]^ which is consistent with our outcomes. In addition to antioxidative stress, iron/heme metabolism is the target mediated by Nrf2. Our results showed that VDR activation upregulated the expression of Nrf2 to inhibit ferroptosis by improving antioxidant capacity and depressing iron content. HO‐1 is one of the main targets of Nrf2 and catalyzes the catabolism of heme into biliverdin, carbon monoxide, and iron, thereby exerting antioxidant, anti‐inflammatory, and anti‐apoptotic effects against diabetic kidney injury.^[^ ^37^, ^38^ ^]^ In this study, both PAR and Fer‐1 could upregulate the expression of HO‐1 in diabetic mice, and this effect was abolished by inhibition of Nrf2. It is interesting that the excess iron content might be derived from the over‐activated HO‐1. Feng et al. stated the expression of and HO‐1 were increased in *db/db* mice, and Fer‐1 treatment inhibited HO‐1 in *db/db* mouse kidneys,^[^ ^39^ ^]^ which is inconsistent with our research findings.^[^ ^13^ ^]^ In this study, we observed that HO‐1 expression was downregulated in DN mice, and most studies proposed that its antioxidant capacity was decreased under the hyperglycemic condition. The cause of the controversial results might be confounding variables in the experimental models or differences in assays.

In conclusion, VDR activation effectively alleviated renal tubular epithelial cell ferroptosis and renal damage upon diabetic insult by enhancing the Nrf2/HO‐1 signaling pathway. Considering that PAR is an active VD analog used for the treatment of kidney diseases, our study provides new insight into the utilization of PAR in DN. Our findings pave the way for developing a feasible therapeutic strategy for ferroptosis‐related chronic kidney diseases.

## Conflict of Interest

The authors declare no conflict of interest.

## Author Contributions

H.W. and X.Y. contributed equally to this work. H.W., X.Y., and Q.F. designed this study, performed the experiments, and made major contributions in writing the manuscript. H.W., X.Y., D.L., Y.Q., J.H., L.Z., S.P., and R.W. participated in collecting data and performing the statistical analysis. Q.F. and Z.L. made substantial contributions to the design and critical revision of the manuscript. All authors have read and agreed to the published version of the manuscript.

## Data Availability

The data that support the findings of this study are available from the corresponding author upon reasonable request.
